# Clinical features of patients with familial Mediterranean fever over 50 years of age: a single-center experience

**DOI:** 10.1007/s11739-026-04272-7

**Published:** 2026-02-16

**Authors:** Sura Nur Baspinar, Feyza Nur Azman Tunc, Berkay Kilic, Mebrure Burcak Yuzbasioglu, Selcan Seven Yenigun, Taha Ayalti, Betul Sahin, Sahar Alizada, Mert Candan, Tugba Bayraktar, Mustafa Furkan Hiyamli, Irem Sena Sarac, Mert Kirman, Baran Can Polat, Serdal Ugurlu

**Affiliations:** 1 Department of Internal Medicine, Cerrahpasa Faculty of Medicine, Istanbul University-Cerrahpasa, Istanbul, Turkey; 2 Cerrahpasa Faculty of Medicine, Istanbul University-Cerrahpasa, Istanbul, Turkey; 3 Division of Rheumatology, Department of Internal Medicine, Cerrahpasa Faculty of Medicine, Istanbul University-Cerrahpasa, 53 Kocamustafapasa Street, Fatih, Istanbul, 34098 Turkey

**Keywords:** Aging, Autoinflammatory diseases, Colchicine, Familial Mediterranean fever

## Abstract

Familial Mediterranean Fever (FMF) is the most common hereditary monogenic autoinflammatory syndrome, with disease activity potentially influenced by aging. This study aimed to evaluate the clinical features, genetic background, treatment patterns, and disease course of FMF in older patients. This retrospective observational cohort study included 343 FMF patients aged 50 years or older. Demographic data, MEFV mutations, attack characteristics, and treatments were analyzed. Attack frequency and visual analog scale (VAS) scores were compared across three time points: before colchicine treatment, after treatment, and in the past year. Patients were stratified by recent attack status, and multivariate logistic regression was used to identify independent predictors of attack activity. The median number of attacks per year decreased from 12 (IQR: 4–24) before treatment to 1 (IQR: 0–4) after treatment, and 0 (IQR: 0–3) in the past year (p < 0.001). Patients with attacks in the past year had higher current and maximum colchicine doses (p = 0.002 and p = 0.02), while those without recent attacks were significantly older (p = 0.005). Multivariate analysis showed that age ≥ 60 years remained associated with a lower likelihood of attacks (OR = 0.60, 95% CI: 0.37–0.98, p = 0.042), as was male sex (p = 0.003). Attack frequency and colchicine requirements were lower in older FMF patients, suggesting milder disease activity in older individuals. These findings highlight the need for additional research into aging-related mechanisms in FMF and may help guide treatment strategies for older patients.

**Supplementary Information:**

The online version contains supplementary material available at 10.1007/s11739-026-04272-7.

## Introduction

Familial Mediterranean fever (FMF) is the most common hereditary monogenic autoinflammatory disease, characterized by short, self-limiting inflammatory episodes that typically resolve within 1–4 days [1]. These episodes often present with fever, serositis (peritonitis, pleuritis, pericarditis), arthritis, and erysipelas-like erythema [2].

FMF is caused by biallelic mutations in the MEFV (Mediterranean fever) gene and follows an autosomal recessive inheritance pattern [3]. The condition predominantly affects individuals of Mediterranean descent, including Armenians, Arabs, and Turks. Among these, the Turkish population exhibits the highest prevalence, estimated to range from 1:150 to 1:1000 [4].

Colchicine remains the mainstay of FMF treatment, with interleukin-1 (IL-1) antagonists reserved for cases that are resistant to or intolerant of colchicine [5]. Early diagnosis and consistent colchicine therapy are critical for preventing FMF-related complications, particularly amyloidosis, which is more common in patients with homozygous M694V mutations [6].

Because FMF is driven by innate immune dysregulation, disease activity may be influenced by age-related immune changes (e.g., immunosenescence) [7]. Previous studies, including Ugurlu et al., have suggested that both attack frequency and colchicine dose requirements decrease with advancing age, raising questions about whether treatment needs or attack patterns change in later adulthood [8].

Evidence regarding FMF disease activity and management in older adults remains limited. In addition, the clinical phenotype in this age group is not well defined. To address this gap, this study aimed to assess the clinical features, genetic background, attack frequency, and treatment profiles of FMF patients aged 50 years and older. By evaluating a large cohort from a single tertiary center, this study seeks to clarify the potential impact of aging on FMF activity and to guide age-adapted management strategies.

## Methods

This retrospective observational cohort study included patients diagnosed with FMF who were treated at Istanbul University-Cerrahpasa, Cerrahpasa Faculty of Medicine, Division of Rheumatology from 2005 to 2020 and who were 50 years or older as of 2022.

The study protocol received approval from the Ethics Committee of Istanbul University-Cerrahpasa, Faculty of Medicine (Approval No: E-83045809-604.01.01.01–51404).

Patients aged 50 and older as of 2022 who met the Tel Hashomer criteria were included [9]. Exclusion criteria were: cognitive impairment, failure to meet diagnostic criteria, refusal to participate, or inability to be contacted due to outdated or missing contact information.

**Fig. 1:**
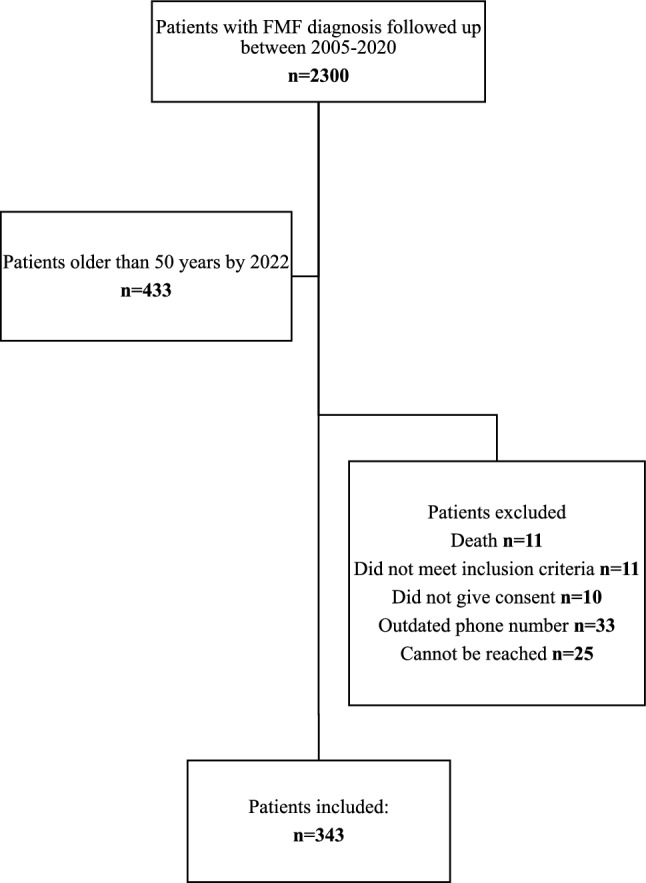
Flow chart of the patient selection. Data extraction is based on patients with FMF diagnosis followed up between 2005 and 2020. Final number of patients included is 343

Of approximately 2,300 FMF patients in the clinic database, 433 were successfully contacted. In October 2022, all reachable patients were contacted via telephone. Information was obtained regarding the date and nature of their most recent FMF attack, the characteristics of the attacks, the Visual Analog Scale (VAS) score for pain during the latest attack, adherence to and response to colchicine, and any adverse events related to treatment. Of the 433 patients contacted, 90 were excluded: 11 had died, 11 did not meet the inclusion criteria, 10 declined participation, 25 had valid contact information but were unreachable, and 33 had outdated contact details. Among the deceased, causes of death included chronic kidney disease (n = 3), COVID-19 (n = 3), coronary artery disease (n = 2), diabetes-related complications (n = 1), and malignancy (n = 1). Ultimately, 343 patients were included in the analysis (Fig. 1).

All patients included in the study were contacted by telephone during the study period to assess disease activity in the past year. Telephone contact was performed even in patients who had recently attended outpatient follow-up visits, in order to identify any FMF attacks that may have occurred after their last clinic visit. For patients who had attended outpatient follow-up within the past year, baseline information on attack frequency and pain severity was obtained from medical records and updated only if a new attack had occurred. For patients who had not attended outpatient follow-up within the past year, representing approximately 30% of the cohort, classification of attack-free status in the past year was based on structured telephone interviews.

Patient records were reviewed to extract data on demographics, age at symptom onset, age at diagnosis, diagnostic delay, follow-up duration, MEFV mutation status, comorbidities, attack frequency, VAS score, treatment history, and biologic therapy usage. Additionally, data on family history, trigger factors, and symptoms during attacks were recorded.

VAS scores were primarily obtained from outpatient medical records, where pain severity during FMF attacks had been routinely documented during clinical follow-up. During the conduct of the study, telephone interviews were used solely to determine whether any new FMF attacks had occurred after the last documented outpatient visit. If no new attack had occurred, previously recorded VAS scores were used without modification. In patients who reported a new attack after the last clinic visit, the VAS score of that attack was obtained through structured telephone interview. Given the retrospective nature of this assessment, patient-reported VAS scores for attacks not contemporaneously documented in medical records may be subject to recall bias.

Attack-related features, VAS scores, and attack frequencies were analyzed across three time points: before colchicine treatment, after the initiation of colchicine, and during the most recent attack. Pre-treatment attacks were defined as those occurring before the effective colchicine dose was administered. Post-treatment attacks referred to those occurring after colchicine initiation and primarily reflected the early treatment phase during which attack frequency and pain severity were routinely documented during outpatient follow-up; this post-treatment period therefore represents a heterogeneous time interval. Characteristics of prior and post-treatment attacks were obtained retrospectively from medical records. For patients who did not experience any attacks within the past year, the most recent documented attack in the medical records was used for analysis. The time interval since the last documented attack varied among patients and was not uniform across the cohort. Attack frequencies were calculated as percentages of the total study cohort, regardless of whether patients had experienced an attack in the prior year.

Pain severity was assessed using the VAS, ranging from 1 (mildest) to 10 (most severe) [10]. Colchicine resistance was defined as ≥ 1 attack per month or persistently elevated acute phase reactants despite six months of maximum tolerated colchicine therapy [11].

Patients were grouped by the presence or absence of at least one M694V mutation to assess genotype–phenotype correlations. Additionally, the cohort was divided into two groups based on whether they had experienced at least one attack within the past year. Comparisons were made between these groups regarding demographic, clinical, genetic, and treatment variables.

### Statistical analysis

All statistical analyses were performed using R software version 4.1.2 in RStudio version 2022.7.1. Descriptive statistics were presented as numbers and percentages for categorical variables, and as mean ± standard deviation (SD) or median with interquartile range (IQR) for continuous variables, depending on distribution. The chi-square test was used for categorical comparisons, and the Mann–Whitney U test was used for non-normally distributed continuous data. ANOVA was used for normally distributed comparisons of more than two groups, while the Kruskal–Wallis test was used for non-normally distributed data. A p-value < 0.05 was considered statistically significant.

Multivariate logistic regression was performed to identify independent factors associated with FMF attack activity in the past year. The dependent variable was at least one FMF attack within the last 12 months. Independent variables included age (categorically analyzed with a ≥ 60-year threshold), gender, disease duration, colchicine resistance, biologic therapy use, and MEFV mutation status. Odds ratios (OR) with 95% confidence intervals (CI) were reported.

## Results

A total of 343 patients with familial Mediterranean fever (FMF) were included in the study (224 females, 119 males). The demographic characteristics are summarized in Table 1. While there was no significant difference in the age at symptom onset between sexes, female patients were diagnosed at a later age (p = 0.006) and experienced a longer diagnostic delay (p = 0.001), whereas male patients had a significantly longer follow-up duration (p < 0.001). Symptom onset after age 40 occurred in 60 patients (17%), and 182 patients (53%) were diagnosed after age 40.

**Table 1:** Demographic characteristics of the patients*

		All (n = 343)	Female (n = 224)	Male (n = 119)
Current age		57.6 ± 6.5	57.4 ± 6.42	58.1 ± 6.65
Age at symptom onset		24 ± 14.4	23.5 ± 14.8	24.8 ± 13.6
Age at diagnosis		40.8 ± 10.8	42 ± 10.6	38.6 ± 10.8
Diagnostic delay		16.8 ± 14.5	18.5 ± 14.8	13.8 ± 13.5
Follow-up duration		16.8 ± 9.79	15.4 ± 8.98	19.4 ± 10.7

*All values are indicated as mean ± SD (years)

Comorbidities were present in 253 patients (74%). The most common were hypertension (43%), type 2 diabetes mellitus (21%), coronary artery disease (12.8%), hypothyroidism (9.6%), and hyperlipidemia (9.3%). Chronic kidney disease was reported in 32 patients (9%), of whom 18 (5.3%) had biopsy-confirmed FMF-related AA amyloidosis. Of these, five patients (1.5%) required dialysis and four (1.2%) had undergone renal transplantation due to amyloidosis. Rheumatologic comorbidities included ankylosing spondylitis (5.8%), rheumatoid arthritis (3.5%), psoriatic arthritis/psoriasis (3.2%), systemic lupus erythematosus (1.7%), and Behçet’s disease (1.2%).

Biologic therapy was administered to 22 patients (6.4%). Indications included colchicine resistance (n = 12), amyloidosis (n = 2), and comorbid inflammatory conditions (n = 8). Among those treated for FMF-related disease, 11 received anakinra and 3 received canakinumab.

MEFV gene mutation analysis was available for 275 patients (80.2%) (Supplementary Table S1). Among them, 217 (78%) carried at least one exon 10 mutation, and 165 (60%) had at least one M694V mutation.

Attack characteristics are detailed in Table 2. Significant differences were noted in the frequency of fever, abdominal pain, arthralgia, arthritis, and chest pain across the three periods (p < 0.001). In comparing the pre-treatment and most recent attack periods, fever, abdominal pain, arthritis, and chest pain significantly decreased (p < 0.001 for all), while myalgia increased (p = 0.01). A similar reduction in fever, abdominal pain, arthralgia, arthritis, and chest pain was observed when comparing the post-treatment period with the most recent attacks (p < 0.001 for all).

**Table 2:** Clinical characteristics of the attacks

Attack Characteristics, n (%)	Before treatment	After treatment	Latest	p-value
Fever	266 (77.6)	253 (73.8)	52 (15)	** < 0.001**
Abdominal pain	293 (85.4)	299 (87.2)	223 (65)	** < 0.001**
Arthralgia	56 (16.3)	82 (23.9)	33 (9.6)	** < 0.001**
Arthritis	87 (25.4)	100 (29.2)	32 (9.3)	** < 0.001**
Chest pain	71 (20)	79 (23)	24 (7)	** < 0.001**
Myalgia	23 (6.7)	34 (9.9)	46 (13.5)	0.14
Erysipelas like erythema	11 (3.2)	12 (3.5)	7 (2)	0.487

Bold values indicate statistical significance (p < 0.05)

The median number of attacks per year declined from 12 (IQR: 4–24) before treatment to 1 (IQR: 0–4) after treatment and to 0 (IQR: 0–3) in the past year. These differences were statistically significant (p < 0.001). Pairwise comparisons showed substantial decreases between the pre-treatment and post-treatment periods (p < 0.001), the pre-treatment period and the past year (p < 0.001). The post-treatment period represented a heterogeneous time interval following colchicine initiation, primarily reflecting the earlier treatment phase, during which attack frequency was routinely documented during outpatient follow-up.

Disease severity, assessed by the VAS score, also improved. The mean VAS score decreased from 8.05 ± 1.78 before treatment to 2.91 ± 2.53 after treatment (p < 0.001), and further to 1.75 ± 2.38 during the most recent attacks (p < 0.001). Each comparison showed a statistically significant reduction in pain severity (p < 0.001).

Table 3 summarizes patients’ initial, maximum, and most recent colchicine doses, treatment compliance, clinical response, dose-skipping behavior, colchicine resistance, and other treatment-related details.

**Table 3:** Treatment details of the patients

Age at the initiation of colchicine treatment, (years), mean ± SD	41.7 ± 10.9
Duration of colchicine treatment (years), mean ± SD	16.0 ± 9.86
The time interval between symptom onset and colchicine initiation (years), mean ± SD	17.7 ± 15.1
Patients using colchicine during the latest follow-up, n (%)	314 (91.5)
Colchicine dose during the latest follow-up (mg/day), mean ± SD	1.29 ± 0.43
Initial colchicine dose (mg/day), mean ± SD	1.37 ± 0.36
Maximum colchicine dose (mg/day), mean ± SD	1.6 ± 0.43
Compliance to colchicine, n (%)	212 (67)
Response to colchicine, n (%)	325 (94.8)
Dose-skipping during colchicine treatment, n (%)	101 (32)
Resistance to colchicine, n (%)	45 (13.1)
Patients using biologic drugs during the latest follow-up, n (%)	22 (6.4)

Patients were stratified according to the presence of at least one M694V mutation, and related clinical features are presented in Supplementary Table S2.

To further explore clinical and genetic factors associated with disease activity, the cohort was stratified by the presence or absence of attacks in the past year (Table 4). Patients who were attack-free were significantly older (p = 0.005) and more likely to be female (p < 0.001). No significant differences were observed between the groups in age at symptom onset, diagnosis age, disease duration, or diagnostic delay. MEFV-negative status was more common in patients with recent attacks (p = 0.016), while exon 10 mutations were more frequent among attack-free patients (p < 0.001). Although the overall prevalence of M694V mutations did not differ, heterozygous M694V mutations were more frequent in the attack-free group (p = 0.006).

**Table 4:** Characteristics of patients who did not experience any attack in the last year and patients who experienced at least one attack in the previous year

	Patients who did not experience attacks (n = 181)	Patients who experienced attacks (n = 162)	p-value
Gender (Female), n (%)	102 (56)	122 (75)	** < 0.001**
Age (years), mean ± SD	58.2 ± 6.43	56.9 ± 6.52	**0.005**
Age at diagnosis (years), mean ± SD	40.8 ± 10.6	40.8 ± 10.9	0.949
Age at symptom onset (years), mean ± SD	24.2 ± 14.2	23.7 ± 14.6	0.336
Diagnostic delay (years), mean ± SD	16.6 ± 14.3	17.1 ± 14.8	0.939
Disease duration (years), mean ± SD	17.4 ± 10.2	16 ± 9.25	0.168
No MEFV mutation, n (%)	8 (4.4)	19 (11.7)	**0.016**
Exon 10 mutation, n (%)	163 (90)	120 (75)	** < 0.001**
At least one M694V mutation, n (%)	92 (50.8)	73 (45)	0.094
M694V heterozygous, n (%)	77 (42.3)	50 (30.9)	**0.006**
FMF in family history, n (%)	124 (68.5)	125 (77.1)	0.367
Colchicine dose during the latest follow-up (mg/day), mean ± SD	1.24 ± 1.0	1.35 ± 0.453	**0.002**
Maximum colchicine dose (mg/day), mean ± SD	1.53 ± 0.375	1.68 ± 0.485	**0.02**
Initial colchicine dose (mg/day), mean ± SD	1.38 ± 0.330	1.37 ± 0.429	0.801
Compliance with colchicine, n (%)	118 (65.2)	94 (58.0)	0.173
Response to colchicine, n (%)	173 (95.5)	152 (93.8)	0.467
Dose-skipping during colchicine treatment, n (%)	44 (24.3)	57 (35.2)	**0.028**
Resistance to colchicine, n (%)	17 (9.4)	28 (17.3)	**0.047**
Treatment with IL-1 inhibitors during the latest follow-up, n (%)	7 (3.9)	15 (9.3)	**0.042**

Bold values indicate statistical significance (p < 0.05)

The mean current and maximum colchicine doses were significantly higher in patients with recent attacks (p = 0.002 and p = 0.02, respectively). In addition, dose skipping, colchicine resistance, and active use of biologic agents were more frequently reported in this group (p = 0.028, p = 0.047, and p = 0.042, respectively).

To investigate the independent role of aging in disease activity, a multivariate logistic regression model was performed using the presence of an attack in the past year as the outcome variable. When age was modeled as a continuous variable, the association with attack occurrence was not statistically significant (p = 0.154). However, when age was analyzed categorically (≥ 60 years vs. < 60 years), patients aged 60 or older had significantly lower odds of experiencing an attack (OR = 0.60, 95% CI: 0.37–0.98, p = 0.042). This effect remained significant after adjustment for sex, colchicine resistance, disease duration, biologic therapy, and MEFV mutation status.

In addition, male sex was independently linked to a lower likelihood of attacks (p = 0.003), whereas biologic therapy correlated with a higher likelihood of attacks (p = 0.016), likely reflecting more severe or treatment-resistant disease. The presence of an M694V mutation exhibited a borderline association with increased attack risk (p = 0.052) (Table 5).

**Table 5:** Logistic regression analysis for attack occurrence in the past year

	Univariate OR (95% CI)	p-value	Multivariate OR (95% CI)	p-value
Age ≥ 60 years	0.62 (0.40–0.95)	0.03	**0.60 (0.37–0.98)**	**0.042**
Male sex	0.48 (0.30–0.75)	< 0.001	**0.52 (0.34–0.80)**	**0.003**
Colchicine resistance	1.65 (1.01–2.70)	0.047	1.58 (1.01–2.55)	**0.047**
Biologic therapy	2.10 (1.10–4.00)	0.02	1.95 (1.13–3.38)	**0.016**
Disease duration	1.00 (0.99–1.01)	0.84	1.00 (0.99–1.01)	0.87
M694V mutation	1.42 (0.98–2.05)	0.06	1.40 (0.99–2.00)	0.052

Multivariate logistic regression analysis evaluating factors associated with FMF attacks in the past year. ORs are presented with 95% confidence intervals

Bold values indicate statistical significance (p < 0.05)

## Discussion

This study evaluated a large cohort of FMF patients aged 50 years and older and provides real-world data on clinical features, genetic background, treatment patterns, and the possible impact of aging on disease activity. Overall, older adults reported fewer attacks in the past year and required lower colchicine doses, suggesting that FMF activity may be associated with different clinical patterns in later adulthood.

Consistent with previous studies, a delay in diagnosis was observed, particularly among female patients. In our cohort, the diagnostic delay was notably longer in women, reflecting a pattern also seen in earlier literature, which attributes this to nonspecific symptom presentation and lower clinical suspicion among physicians [12, 13].

The MEFV mutation most commonly observed in this cohort was M694V, both in heterozygous and homozygous forms, along with other pathogenic variants such as M680I and V726A. As reported in previous literature, M694V mutations have been associated with earlier onset and more severe disease, including a higher risk of renal amyloidosis [11]. In our analysis, patients carrying at least one M694V mutation were diagnosed at a younger age and were more likely to have a family history of FMF, yet mutation status was not associated with attack frequency or colchicine dose in later life.

The absence of an association between M694V mutation status and current disease activity should be interpreted in the context of disease evolution over time. While M694V, particularly in homozygous form, is a well-established determinant of earlier disease onset and greater severity, its influence on ongoing inflammatory activity may diminish with advancing age. This pattern is consistent with age-related immunomodulation, whereby immunosenescence and reduced innate immune responsiveness may attenuate inflammatory manifestations over time, even in genetically predisposed individuals. Accordingly, M694V may primarily influence the initial disease course, whereas aging-related immune remodeling may play a greater role in shaping disease activity in later life, leading to a weakening of genotype–phenotype associations in older patients rather than a contradiction of established genetic risk models. In addition, this lack of association between M694V mutation status and current disease activity may also be partly influenced by the relatively lower proportion of homozygous M694V patients in the cohort.

Although the presence of an M694V mutation showed a borderline association with increased attack risk in multivariate analysis, this did not reach statistical significance, supporting the interpretation that genotype alone does not independently determine current disease activity in later life.

Age emerged as an important factor associated with current disease activity. In the multivariate logistic regression, patients aged 60 years or older had significantly lower odds of experiencing an attack in the past year, independent of gender, colchicine resistance, disease duration, biologic use, and genotype. Given the retrospective observational design, this finding should be interpreted as an association rather than a causal effect, and the magnitude of this association was modest, warranting cautious interpretation. One possible explanation may involve age-related immunomodulation—including immunosenescence and reduced innate immune responsiveness—which could contribute to a blunted inflammatory phenotype in older patients. Although biologic use and colchicine resistance were more common among those with recent attacks, older individuals overall were more frequently attack-free.

Subgroup analysis was consistent with this age-related shift in disease expression. Patients without attacks in the past year were significantly older and more frequently carried heterozygous exon 10 mutations, including M694V. They also required lower colchicine doses and showed fewer features of treatment resistance. Although a greater proportion of females appeared in the attack-free group, multivariate analysis demonstrated that male sex was independently associated with a lower attack risk, indicating that unadjusted sex differences were likely influenced by other clinical variables.

The higher frequency of heterozygous M694V variants among older, attack-free patients should be interpreted cautiously and likely reflects age-related immunomodulation and phenotypic selection over time rather than a protective genetic effect. Accordingly, the apparent weakening of genotype–phenotype associations in older patients likely reflects disease evolution across the lifespan rather than a contradiction of established genetic risk models. In this context, heterozygous carriers may be overrepresented among older, attack-free patients because they tend to have a milder initial disease course and are therefore more likely to reach older age with lower residual inflammatory activity.

The concept of “inflammaging”—a chronic, low-grade inflammatory state associated with aging—offers a possible mechanistic explanation. While aging is often associated with increased levels of proinflammatory cytokines (e.g., IL-6, IL-1β, TNF-α), it also induces counter-regulatory, anti-inflammatory pathways and cellular senescence [14–19]. These opposing forces may contribute to immune system remodeling and a blunted inflammatory response in older FMF patients. This dual effect may explain the observed reduction in attack frequency and colchicine requirements, without suggesting true disease remission.

Although colchicine is typically recommended as lifelong therapy, EULAR guidelines allow dose reduction in patients who remain attack-free with normalized inflammatory markers for at least five years [11]. Given the lower disease activity observed in patients aged 60 and above, our findings support the potential for age-tailored colchicine dose adjustment in selected older FMF patients.

This study has several limitations. Its retrospective design introduces risks of recall bias, particularly for patient-reported pain severity assessed via telephone interview when attacks occurred after the last outpatient visit, and approximately 30% of patients had not attended outpatient follow-up within the past year. In this subgroup, classification as attack-free was based on structured telephone interviews rather than contemporaneous laboratory assessment. Although acute-phase reactants such as C-reactive protein (CRP) and erythrocyte sedimentation rate (ESR) were available from routine follow-up visits and previous attacks, these parameters were not systematically available for the most recent attacks assessed by telephone in patients who did not attend in-person follow-up, limiting objective confirmation of inflammatory quiescence and colchicine resistance. However, many were successfully contacted by telephone, and some reported being asymptomatic for several years, explaining their lack of follow-up and supporting the reliability of the attack information obtained. In addition, the timing of the “last remembered attack” among attack-free patients was heterogeneous and based on patient recall, which may have introduced variable recall bias.

In addition, this was a single-center retrospective study conducted at a tertiary referral center in a country with a high prevalence of FMF. Such a setting may introduce selection bias, as patients with more severe disease phenotypes, complications such as amyloidosis, or treatment-resistant disease may be overrepresented. Conversely, patients with the most severe disease may have been lost to follow-up or died before inclusion, leading to potential survivor bias, particularly among older age groups. These factors may limit the generalizability of the findings to broader FMF populations, especially those managed in non-tertiary care settings or in regions with lower disease prevalence.

Despite these limitations, the study possesses notable strengths: all eligible patients were individually contacted, allowing confirmation of recent attack history; genetic and clinical data were detailed and comprehensive; and the inclusion of a large number of older adults—an underrepresented population in FMF literature—provides valuable insight into disease characteristics in later adulthood.

## Conclusions

In this large cohort of FMF patients aged 50 years and older, advancing age was associated with fewer attacks, lower pain severity, and reduced colchicine requirements. Age ≥ 60 years showed a statistically significant but modest association with being attack-free after adjustment for relevant clinical factors. These findings indicate an association rather than a causal effect and should be interpreted within the limitations of the retrospective observational study design. Although lifelong colchicine therapy is recommended, our findings highlight the importance of periodic reassessment and individualized treatment decisions in older patients. Further prospective studies are needed to clarify the mechanisms underlying age-related changes in FMF and to define their implications for long-term management.

## Supplementary Information

Below is the link to the electronic supplementary material.

Supplementary file1 (DOCX 20 KB)

## Data Availability

The datasets analyzed during the current study are not publicly available due to patient confidentiality but are available from the corresponding author on reasonable request.
