# Supplementary material for: Clinical features of patients with familial Mediterranean fever over 50 years of age: a single-center experience
Source: Intern Emerg Med. 2026 Feb 16;21(3):1011–8. doi: 10.1007/s11739-026-04272-7 (PMC13144219; doi:10.1007/s11739-026-04272-7)
Supplement: Supplementary file 1 — Supplementary file1 (DOCX 20 KB) [file 11739_2026_4272_MOESM1_ESM.docx]

**Supplementary Table S1. MEFV gene status**

| MEFV gene status, n (%) | Patients with genetic results (n=275) |
| --- | --- |
| Exon 10 Mutation |  |
| M694V Homozygous | 38 (13.8) |
| M680I Homozygous | 10 (3.6) |
| V726A Homozygous | 5 (1.8) |
| M694V Heterozygous | 127 (46.1) |
| M680I Heterozygous | 40 (14.5) |
| V726A Heterozygous | 44 (16) |
| At least one M694V Mutation | 165 (60) |
| At least one Exon 10 Mutation | 217 (78) |
| Exon 2 Mutation |  |
| R202Q Homozygous | 7 (2.5) |
| E148Q Homozygous | 1 (0.3) |
| R202Q Heterozygous | 46 (16.7) |
| E148Q Heterozygous | 28 (10.1) |
| At least one Exon 2 Mutation | 78 (28) |
| No mutation | 27 (10) |
| Unknown | 68 (19.8) * |

*Percentage of all patients

**Supplementary Table S2.** Comparison of patients with at least one M694V mutation (Group 1) with patients without M694V mutation (Group 2)

|  | Patients with at least one M694V Mutation  (n=165) | Patients without M694V Mutation  (n=110) | p-value |
| --- | --- | --- | --- |
| Gender (Female), n (%) | 113 (68) | 76 (69) | 0.916 |
| Age at symptom onset (years), mean ± SD | 24.8 ± 14.5 | 24.7 ± 14.6 | 0.605 |
| Age at diagnosis (years), mean ± SD | 41.8 ± 11.1 | 42.0 ± 9.54 | **<0.001** |
| Disease duration (years), mean ± SD | 16.0 ± 9.56 | 14.2 ± 7.67 | 0.733 |
| FMF in family history, n (%) | 135 (81.8) | 67 (60.9) | **0.001** |
| Duration of attacks (days), mean ± SD | 2.84 ± 1.56 | 2.83 ± 1.45 | 0.625 |
| Number of attacks before treatment (per year) * | 12 (4-24) | 12 (4-24) | 0.353 |
| Number of attacks after treatment (per year) * | 1 (0-4) | 1 (0-4) | 0.291 |
| Number of the latest attacks (per year) * | 0 (0-2) | 0 (0-3) | 0.255 |
| Colchicine dose during the latest follow-up (mg/day), mean ± SD | 1.31 ± 0.446 | 1.27 ± 0.437 | 0.399 |
| Initial colchicine dose (mg/day), mean ± SD | 1.37 ± 0.371 | 1.34 ± 0.383 | 0.549 |
| Maximum colchicine dose (mg/day), mean ± SD | 1.58 ± 0.386 | 1.61 ± 0.463 | 0.515 |
| Compliance with colchicine, n (%) | 114 (72) | 63 (64) | 0.16 |
| Response to colchicine, n (%) | 153 (92.7) | 106 (96.3) | 0.208 |
| Dose-skipping during colchicine treatment, n (%) | 43 (27.3) | 31 (31.6) | 0.46 |
| Resistance to colchicine, n (%) | 25 (38.4) | 21 (19.1) | 0.362 |
| Treatment with IL-1 inhibitors during the latest follow-up, n (%) | 13 (7.8) | 9 (8.1) | 0.415 |

*presented in median (IQR).
